# Neurofeedback Training Modulates Brain Functional Networks and Improves Cognition in Amnestic Mild Cognitive Impairment Patients Aged 60–70 Years

**DOI:** 10.3390/brainsci15111243

**Published:** 2025-11-19

**Authors:** Rui Su, Xin Li, Ping Xie, Yi Yuan

**Affiliations:** 1 School of Medical Imaging, Hebei Medical University, Shijiazhuang 050017, China; surui065@hebmu.edu.cn; 2 Hebei Provincial Key Laboratory of Medical Imaging Science, Shijiazhuang 050017, China; 3 Measurement Technology and Instrumentation Key Laboratory of Hebei Province, Qinhuangdao 066004, China; pingx@ysu.edu.cn (P.X.); yuanyi513@163.com (Y.Y.); 4 School of Electrical Engineering, Yanshan University, Qinhuangdao 066004, China

**Keywords:** amnestic mild cognitive impairment, neurofeedback training, electroencephalography, high-order network, low-order network, dynamic functional connectivity

## Abstract

**Background/Objectives**: Amnestic mild cognitive impairment (aMCI) represents a transitional stage between normal aging and dementia, constituting a critical intervention window for Alzheimer’s disease (AD). As a non-invasive intervention, neurofeedback training (NFT) has demonstrated potential in ameliorating cognitive deficits and clinical symptoms in aMCI patients; however, its mechanistic effects on functional brain connectivity remain inadequately elucidated. **Methods**: This study employed low- and high-order functional analytical approaches to comprehensively investigate the effects of NFT on dynamic brain functional networks in aMCI. **Results**: Our findings revealed that following NFT, aMCI patients exhibited enhanced connectivity strength, global efficiency, and nodal characteristics within the delta band, whereas connectivity was generally attenuated in the theta, alpha, and beta bands. Dynamic network analysis indicated increased entropy in short-time windows. Cognitive assessments showed a significant short-term improvement in MoCA scores among 92.9% of participants. **Conclusions**: These results suggest that NFT effectively remodels brain network activity patterns in aMCI patients, thereby facilitating cognitive improvement. These findings provide preliminary insights into the brain network mechanisms underlying NFT-mediated cognitive enhancement in aMCI.

## 1. Introduction

Alzheimer’s Disease (AD) represents a global public health crisis, characterized by a well-defined staging of its pathological progression [1,2,3]. Amnestic Mild Cognitive Impairment (aMCI), serving as a transitional stage between normal aging and AD, is characterized by selective decline in memory function.Its annual conversion rate to AD is significantly higher than that of the healthy elderly population [4,5]. Consequently, exploring safe and effective interventions for aMCI patients within this age group and elucidating their neuromodulatory mechanisms are of critical importance for the early prevention and treatment of AD [6,7].

Current therapeutic strategies for cognitive dysfunction are primarily categorized into pharmacological and non-pharmacological interventions. Pharmacological approaches, such as cholinesterase inhibitors, are widely used in clinical practice; however, their efficacy is limited and often accompanied by significant side effects [8,9]. In contrast, non-pharmacological interventions have garnered increasing attention due to their favorable safety profiles and greater accessibility. Among these, electroencephalogram (EEG)-based neurofeedback training (NFT)—a non-invasive neuromodulation technique—aims to enhance an individual’s ability to voluntarily regulate specific neural oscillations by providing real-time feedback on EEG activity [10,11,12]. Emerging evidence suggests that NFT may improve memory encoding and executive function in individuals with cognitive impairment, potentially through the modulation of neural oscillatory patterns [13]. Faucounau et al. [14] confirmed the efficacy of cognitive interventions in patients with mild cognitive impairment (MCI). Israsena et al. [15] demonstrated that a targeted NFT protocol designed to guide attention significantly enhanced cognitive performance in participants. Furthermore, Alatorre-Cruz et al. [16] provided evidence supporting the potential of NFT in preventing cognitive decline in healthy older adults. Collectively, these findings underscore the important role of NFT in the regulation of cognitive function.

The cognitive benefits of NFT are closely associated with its modulation of brain functional states [17,18]. EEG-based NFT studies primarily employ nonlinear dynamical analysis methods to reveal the dynamic characteristics of brain activity during training [19,20]. Liu et al. [21] demonstrated that NFT significantly increases the peak frequency of the alpha band; Suhail et al. [22] observed significant alterations in EEG rhythms among MCI patients following NFT through power spectral analysis. These findings suggest that NFT may enhance cognitive function by modulating neural oscillatory activities in specific frequency bands.

Recent studies have proposed that aMCI represents a transitional “disconnection syndrome,” where clinical symptoms may be linked to the disruption of functional connectivity between brain regions, potentially induced by amyloid-beta (Aβ) deposition [23,24]. This pathological framework provides a novel perspective for understanding the mechanisms of NFT: it is plausible that NFT ameliorates cognitive impairment by reorganizing functional network connectivity. However, the specific processes through which NFT modulates brain network properties in aMCI patients and thereby influences cognitive function remain poorly understood.

Neurofeedback Training has been shown to improve cognitive function in patients with aMCI. However, the neural mechanisms through which NFT modulates cognitive function in aMCI remain unclear, and a reliable quantitative evaluation framework is still lacking. This study employs a systematic research approach, integrating low- and high-order brain network analyses, dynamic functional connectivity assessments, and other multi-dimensional network characteristics, combined with clinical neuropsychological evaluations, to investigate the brain network mechanisms underlying NFT-induced cognitive improvement in aMCI patients.

## 2. Materials and Methods

### 2.1. Data Source

A total of 28 individuals diagnosed with aMCI were recruited for this study. The cohort consisted of 16 males and 12 females, all aged 60–70 years, with educational durations spanning 10 to 15 years, as shown in Table 1. Participant selection adhered strictly to the Petersen criteria under neurological supervision [25]. Inclusion requirements comprised: (1) self-reported memory impairment; (2) cognitive test scores falling at least 1.5 standard deviations below demographically adjusted norms; (3) largely intact or only mildly affected daily functioning; (4) absence of a dementia diagnosis as defined by DSM-IV; (5) a Clinical Dementia Rating (CDR) global score of 0.5; and (6) education-adapted scores on both the Mini-Mental State Examination (MMSE; illiterate: ≥19, primary: ≥22, secondary or above: ≥24) and the Montreal Cognitive Assessment (MoCA; illiterate: ≥13, primary: ≥19, secondary or above: ≥24).

Exclusion criteria involved: (1) MCI of vascular, traumatic, or secondary origin (such as resulting from neurological conditions, metabolic disorders, or infections); (2) major psychiatric illness or depressive symptoms (indicated by a Hamilton Depression Rating Scale score exceeding 24); (3) substantial vision or hearing loss; and (4) factors precluding MRI safety or compliance, including metal implants or claustrophobia. Ethical approval for this research was obtained from the Ethics Committee of Yanshan University.

EEG data were acquired using the NT9200 system (Beijing Zhongke Xintuo Technology Co., Ltd., Beijing, China) with 16 electrodes. The data employed in this study were 16-channel EEG recordings obtained using the international 10–20 system. The 16 channels were FP1, FP2, F3, and F4 in the frontal lobe, F7, T3, and T5 in the left temporal lobe, F8, T4, and T6 in the right temporal lobe, C3, C4, P3, and P4 in the parietal lobe, and O1, and O2 in the occipital lobe. Signals were sampled at 1000 Hz, with electrode impedance kept under 5 kΩ. Participants were instructed to remain still and awake with closed eyes in a quiet setting during recording. Artifacts from head motion, jaw clenching, or facial movements were minimized through instruction and monitoring. All recordings followed a standardized protocol lasting no less than 5 min per subject. This study received ethics approval from the Ethics Review Committee of Yanshan University (ID: 2022008).

### 2.2. Data Preprocessing

The acquisition of EEG signals is frequently contaminated by diverse forms of noise, including power-line interference, ocular artifacts, and electromyographic (EMG) contamination. Preprocessing is therefore critical for improving signal integrity. Initially, raw EEG recordings were downsampled from 1000 Hz to 128 Hz to alleviate computational burden without compromising relevant neural information. A notch filter was then implemented to suppress 50 Hz AC interference. Further removal of physiological artifacts—such as those arising from eye movements and muscle contractions—was performed using Independent Component Analysis (ICA) to identify and exclude components associated with artifacts. Segments exhibiting prominent motion artifacts were also discarded based on visual scrutiny to enhance data trustworthiness [26]. The cleaned signals were subsequently subjected to wavelet decomposition with a Daubechies (db4) basis, segregating them into four standard frequency bands: delta (1–4 Hz), theta (4–8 Hz), alpha (8–13 Hz), and beta (13–30 Hz). This comprehensive procedure significantly enhanced the overall signal-to-noise ratio of the EEG data.

### 2.3. Neurofeedback Training Protocol

**Figure 1:**
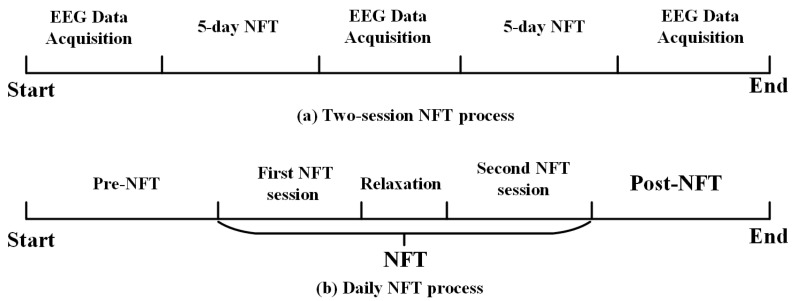
NFT flow chart.

Participants completed two NFT cycles, each spanning five days (Figure 1a), amounting to a total of ten training days per individual. Each cycle is separated by one day. As depicted in Figure 1b, two sessions were administered daily. The rest duration between individual training sessions is one hour. The training game was completed using the “Mind-Force Ant” game, which is controlled by mental focus. At the beginning of the game, the system provides food corresponding to the player’s relaxation index—the higher the value, the fresher the food. The game includes both a relaxation mode and a focus mode, which alternate during gameplay. Participants can control the ant’s movement speed, with the objective of pushing the food to the endpoint or continuing until the time limit (3 min) is reached, at which point the neurofeedback training session ends. EEG recordings were obtained at three time points: prior to training, after the first cycle, and immediately following the second cycle.

### 2.4. Construction of Brain Functional Networks

Construction of Low- and High-Order Networks

**Figure 2:**
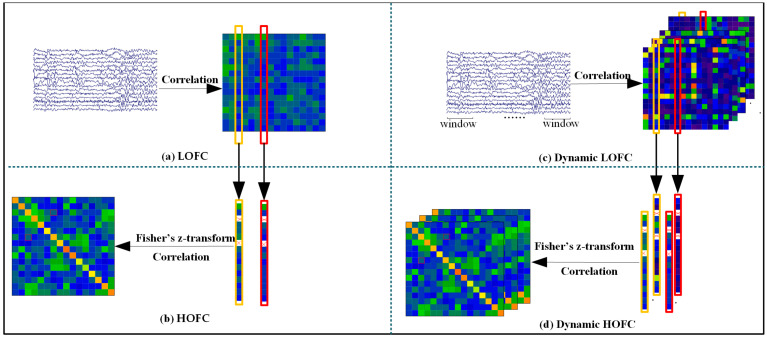
Construction of low-order (**a**) and high-order (**b**) networks. Building dynamic low-order (**c**) and dynamic high-order (**d**) networks.

The construction procedures for both low-order functional connectivity (LOFC) and high-order functional connectivity (HOFC) matrices are illustrated in Figure 2a,b, respectively. First, the Hilbert transform was applied to the EEG signals to obtain their amplitude envelopes. The LOFC matrix was derived by computing the absolute Pearson correlation coefficients between each pair of envelope signals. Subsequently, self-connections were removed from each column of the LOFC matrix.

Each column of the resulting matrix was then subjected to Fisher’s z-transform to improve normality. The high-order functional network was constructed by computing Pearson correlations between every pair of the transformed columns, as depicted in Figure 2b.

Given that EEG signals were analyzed across four frequency bands in this article, four distinct HOFC matrices were generated for each participant.

A multi-level analysis of network characteristics was conducted in participants with aMCI, encompassing both global-level metrics (global efficiency and clustering coefficient) and nodal-level metrics (nodal efficiency and nodal clustering coefficient).

Nodal efficiency quantifies the efficiency of information exchange at a specific node within the network. It is defined as:

(1) $$NE_{i}=\frac{1}{\left(N-1\right)}\sum_{i\neq j}^{N}\frac{1}{d_{ij}}$$

where $d_{ij}$ represents the shortest path length between nodes i and j, and N denotes the total number of nodes in the network.

Global efficiency serves as a key metric reflecting the integrated information transfer capacity of complex brain networks, indicative of their parallel processing capability. It is formally defined as follows:

(2) $$GE=\frac{1}{N}\sum_{i=1}^{N}NE_{i}$$

The nodal clustering coefficient quantifies the extent of interconnectivity between a given node and its immediate neighbors in the network. It is defined as:

(3) $$NCC_{i}=\frac{\sum_{k\neq i}\sum_{i\neq i,i\neq k}w_{ik}w_{i}w_{ij}}{\sum_{k\neq i}\sum_{i\neq i,i\neq k}w_{ik}w_{ij}}$$

where w represents the weight between node i and node j.

The average clustering coefficient is defined as the average of all nodal clustering coefficients across the network:

(4) $$C_{ave}=\frac{1}{N}\sum_{i=1}^{N}NCC_{i}$$

Construction of Dynamic High- and Low-Order Functional Networks

The detailed procedure for constructing the dynamic high-order and low-order functional brain networks is illustrated in Figure 2c,d. For each of the four frequency bands (delta, theta, alpha, and beta), dynamic connectivity matrices were generated using multiple window lengths, resulting in multiple segmented EEG data sets corresponding to each window duration.

This study examined four distinct frequency bands: delta, theta, alpha, and beta. For each band, seven window lengths were applied: the minimum period corresponding to the reciprocal of the center frequency of the band ($1/f_{\min}$), 1 s, 2 s, 4 s, 6 s, 8 s, and 10 s. All windows were non-overlapping to ensure temporal independence of the computed connectivity estimates.

State entropy analysis was employed to characterize the state transitions between integrated and segregated configurations of the dynamic high-order and low-order functional networks. In this framework, the integrated network state was encoded as 1, while the segregated state was encoded as 0. This encoding scheme gives rise to four possible state transition patterns: 00, 01, 10, and 11. The state entropy was subsequently computed for each of these transition patterns to quantify the temporal variability and predictability of network reorganization.

(5) $$NSE=-\frac{1}{log\left(M\right)}\sum_{i=1}^{M}p_{i}log\left(p_{i}\right)$$

where represents the number of distinct transition patterns. In this study, M = 4. Let $p_{i}$ denote the occurrence probability of the *i*-th pattern. The normalized entropy is then defined as:

### 2.5. Statistical Analysis

All statistical analyses were performed using SPSS (SPSS, Chicago, IL, USA) and MATLAB (version 2018b, MathWorks Inc., Natick, MA, USA) software, with paired-samples *t*-tests employed to assess differences in brain network properties of patients with aMCI pre- and post- NFT. Statistical significance was denoted by the symbols “*”, “**”, and “***”, corresponding to *p* < 0.05, *p* < 0.01, and *p* < 0.001, respectively, in line with standard statistical conventions.

The analyzed brain network properties covered 4 frequency bands (delta band: 1–4 Hz, theta band: 4–8 Hz, alpha band: 8–13 Hz, beta band: 13–30 Hz) and multiple dimensions. Specifically, for both LOFC networks and HOFC networks, the analyzed metrics included connectivity strength, global efficiency, average clustering coefficient, as well as nodal metrics (nodal efficiency, nodal clustering coefficient) in the frontal, occipital, parietal, and temporal lobes; for dynamic networks, state entropy was evaluated under 7 window lengths (minimum period of the center frequency of each band, 1 s, 2 s, 4 s, 6 s, 8 s, 10 s).

The visualization of brain functional network connectivity was achieved using the BrainNet Viewer toolbox (Version 1.7). The visualization results featured clear spatial localization (16 electrode positions labeled in accordance with the International 10–20 system, with the frontal lobe orientation clearly specified) and unified color coding (adopting a 0–0.6 scale, where dark blue represents a connectivity strength of 0 and dark red represents a connectivity strength of 0.6), providing an intuitive visual representation of brain network characteristics.

## 3. Results

### 3.1. Low-Order Functional Brain Network Connectivity and Its Characteristics

**Figure 3:**
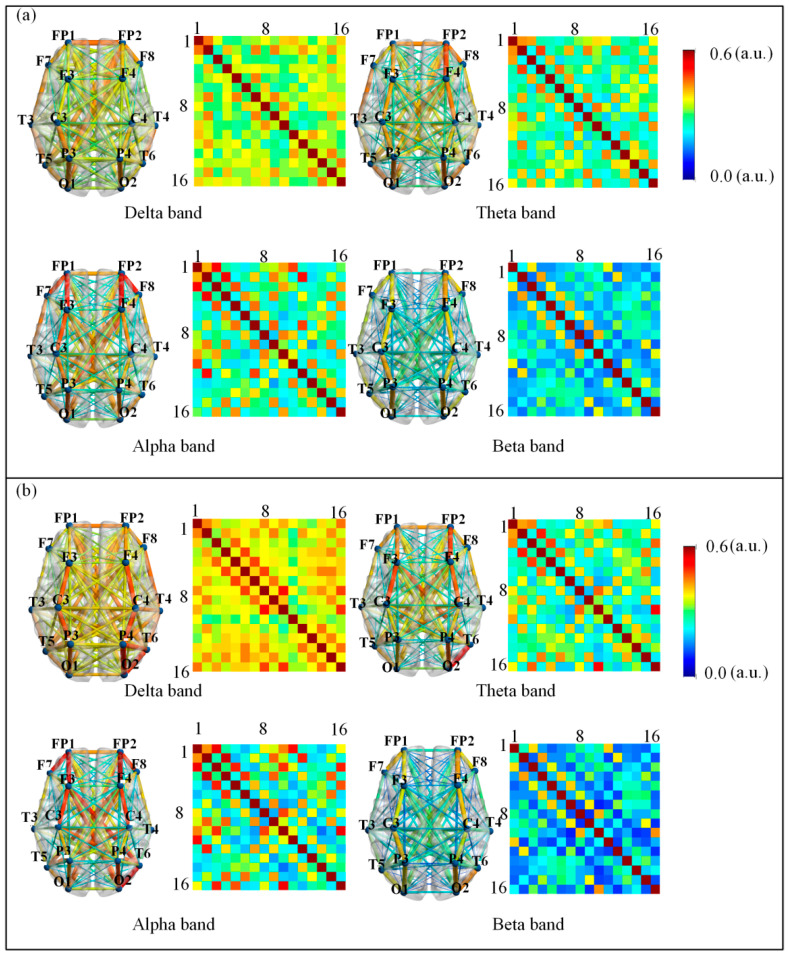
(**a**) Connection diagram of LOFC network before NFT and (**b**) connection diagram of LOFC network after NFT. The color-block maps depict the connection strengths across four distinct frequency bands in aMCI patients pre- and post-NFT. To facilitate comparison of inter-band connectivity differences pre- and post-training, eight color-block maps and eight visual connectivity diagrams were generated (left subplots for each frequency band). The color scale range was set from 0 to 0.6, with deep blue representing 0 and deep red representing 0.6. The 16 channel locations are annotated, respectively, within the color-block maps.

We analyzed the differences in LOFC network strength across four frequency bands (delta, theta, alpha, and beta) pre- and post- NFT in individuals with aMCI. As shown in Figure 3, the connectivity strength for each frequency band is represented using colormaps. To facilitate comparison between pre- and post-NFT states, eight colormaps and eight corresponding connectivity visualization diagrams (left subplot in each frequency panel) are provided. The color scale ranges from 0 to 0.6, with dark blue indicating 0 and dark red indicating 0.6. The colormaps display the positions of the 16 channels, and the connectivity diagrams indicate the frontal lobe orientation. Visual inspection of Figure 3 suggests differences between pre- and post-training colormaps and connectivity diagrams. Specifically, the delta band colormap appears darker after NFT, indicating stronger connectivity. No obvious trends were observed in the other bands based solely on visual comparison. We further compared the mean LOFC network connectivity strength between pre- and post-NFT within each frequency band. The results showed that in the delta band, the mean connectivity strength was lower before training (mean = 0.3818) than after (mean = 0.4174). In contrast, the theta band exhibited higher mean connectivity before training (mean = 0.3476) compared to after (mean = 0.3423). Similarly, the alpha band showed higher pre-training connectivity (mean = 0.3476) than post-training (mean = 0.3414), and the beta band also demonstrated higher pre-training connectivity (mean = 0.2748) relative to post-training (mean = 0.2591). These findings indicate that NFT led to an increase in mean LOFC network connectivity in the delta band, while a decrease was observed in the theta, alpha, and beta bands. Overall, the results suggest that NFT induces changes in low-order functional brain network connectivity in individuals with aMCI.

**Figure 4:**
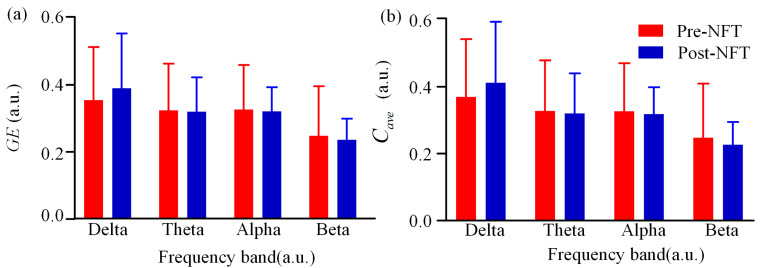
(**a**) GE and (**b**) cave of LOFC network. Red indicates the eigenvector centrality of the LOFC network in aMCI patients before NFT, while blue indicates that after NFT.

We analyzed the global efficiency and average clustering coefficient of the LOFC network across four frequency bands (delta, theta, alpha, and beta) pre- and post- NFT in participants with aMCI. As shown in Figure 4, the values corresponding to the pre-training and post-training conditions are represented in red and blue, respectively. Visual inspection of Figure 4 suggests that in the delta band, both global efficiency and average clustering coefficient were higher after NFT compared to before. In the other frequency bands, however, both metrics exhibited lower values after training. Statistical comparisons of these global network properties pre- and post- NFT revealed no significant differences in any of the frequency bands (*p* > 0.05).

**Figure 5:**
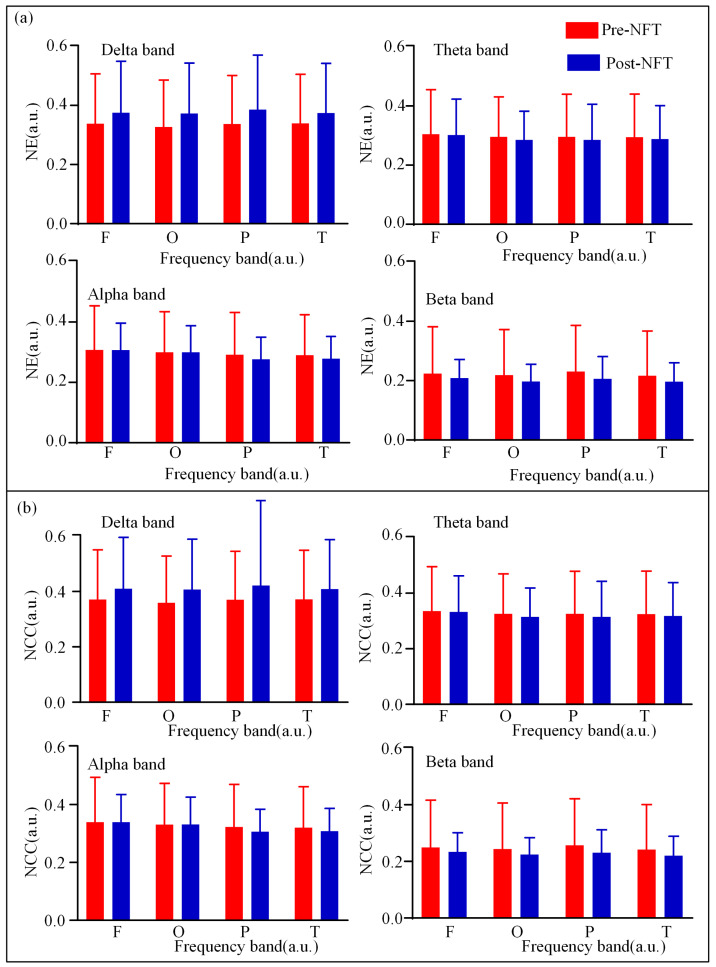
(**a**) NE and (**b**) NCC of LOFC network. Red indicates the eigenvector centrality of the LOFC network in aMCI patients before NFT, while blue indicates that after NFT.

We analyzed the nodal characteristics (Nodal Efficiency, NE, and Nodal Clustering Coefficient, NCC) of the LOFC network across different brain regions (frontal, occipital, parietal, and temporal lobes) pre- and post- NFT in individuals with aMCI. As illustrated in Figure 5, the pre-training and post-training values are depicted in red and blue, respectively. Visual observation of Figure 5 suggests that in the delta band, nodal feature values after NFT were higher across all brain regions compared to those before training. Conversely, in the beta band, the values decreased in all regions following training. Differences in other frequency bands were less pronounced. Statistical comparison of regional nodal characteristics pre- and post- NFT revealed no significant differences in any of the frequency bands across all brain regions (*p* > 0.05). A further quantitative assessment of the nodal characteristics of the LOFC network was conducted. Detailed data for the NE and NCC across different frequency bands and brain regions are presented in Table A1, Table A2, Table A3 and Table A4, located in Appendix A.

### 3.2. High-Order Functional Network Connectivity and Its Characteristics

**Figure 6:**
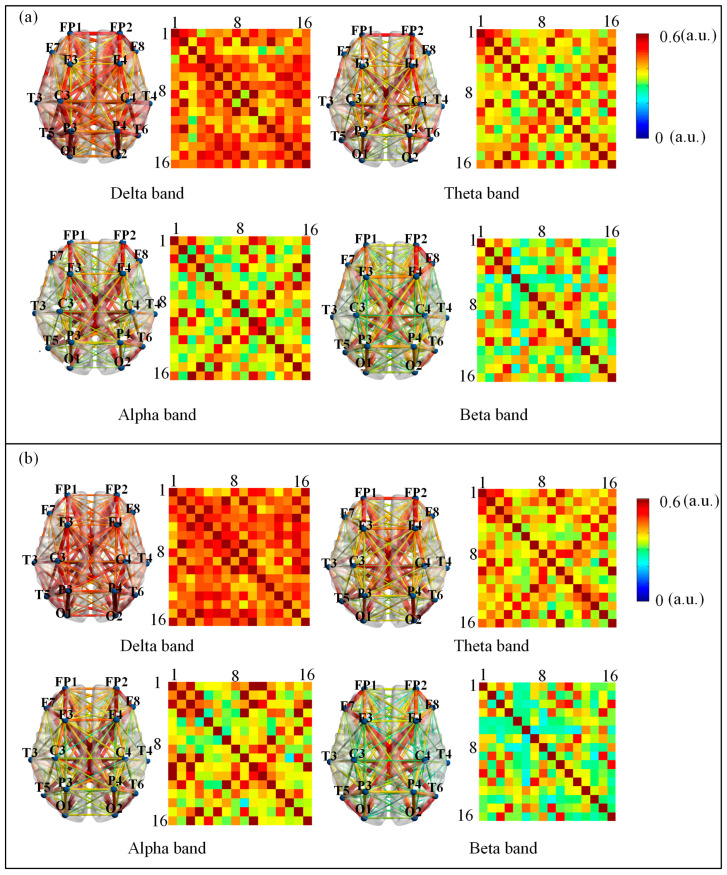
Connection diagram of HOFC network (**a**) pre- and (**b**) post-NFT. The color-block maps depict the connection strengths across four distinct frequency bands in aMCI patients pre- and post- NFT. To facilitate comparison of inter-band connectivity differences pre- and post-training, eight color-block maps and eight visual connectivity diagrams were generated (left subplots for each frequency band). The color scale range was set from 0 to 0.6, with deep blue representing 0 and deep red representing 0.6. The 16 channel locations are annotated, respectively, within the color-block maps.

We analyzed the differences in HOFC network strength across four frequency bands (delta, theta, alpha, and beta) pre- and post- NFT in individuals with aMCI. As illustrated in Figure 6, the connectivity strength for each frequency band is represented using colormaps. To facilitate comparison between pre- and post- NFT conditions, eight colormaps and eight corresponding connectivity visualization diagrams (left subplot in each frequency panel) are provided. The color scale ranges from 0 to 0.6, with dark blue indicating 0 and dark red indicating 0.6. The colormaps display the positions of the 16 channels, and the connectivity diagrams indicate the frontal lobe orientation. Visual inspection of Figure 6 suggests differences between the pre- and post-training colormaps and connectivity diagrams. Specifically, the delta band exhibits a darker colormap after NFT, indicating stronger connectivity, whereas no obvious trends were observed in the other bands based solely on visual assessment. Therefore, we further compared the mean HOFC strength pre- and post- NFT within each frequency band. The mean connectivity values before training were as follows: delta band (mean = 0.4951), theta band (mean = 0.4451), alpha band (mean = 0.4310), and beta band (mean = 0.4015). After training, the mean values were: delta band (mean = 0.5112), theta band (mean = 0.4472), alpha band (mean = 0.4450), and beta band (mean = 0.3864). Comparison of these results indicates that, in the delta band, the mean connectivity strength after NFT was higher than that before training. In contrast, the mean connectivity strength decreased after training in the other three bands. A consistent pattern was observed between the effects of NFT on LOFC and HOFC networks: in the delta band, the mean connectivity strength increased after training, while in the other bands, it decreased.

**Figure 7:**
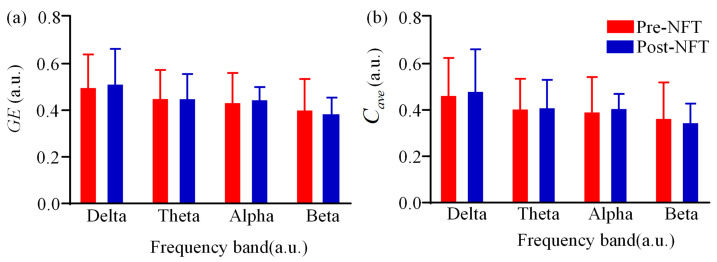
(**a**) GE and (**b**) cave of HOFC network. Red indicates the eigenvector centrality of the LOFC network in aMCI patients before NFT, while blue indicates that after NFT.

We analyzed the differences in global efficiency and average clustering coefficient of the HOFC network across four frequency bands (delta, theta, alpha, and beta) pre- and post- NFT in individuals with aMCI. As shown in Figure 7, the pre-training and post-training values are represented in red and blue, respectively. Visual inspection of Figure 7 suggests that in the delta and alpha bands, both global efficiency and average clustering coefficient were higher after NFT compared to the pre-training values. In contrast, both metrics exhibited a decrease in the beta band following training. For the theta band, the bar plot did not allow clear visual discrimination of differences. Further comparison of specific numerical values revealed that in the theta band, the mean global efficiency was 0.4541 before training and 0.4539 after training, indicating a slight decrease. Conversely, the mean average clustering coefficient increased from 0.4085 before training to 0.4134 after training. Statistical analysis showed no significant differences in either global efficiency or average clustering coefficient between pre- and post- NFT conditions in any of the frequency bands (*p* > 0.05).

**Figure 8:**
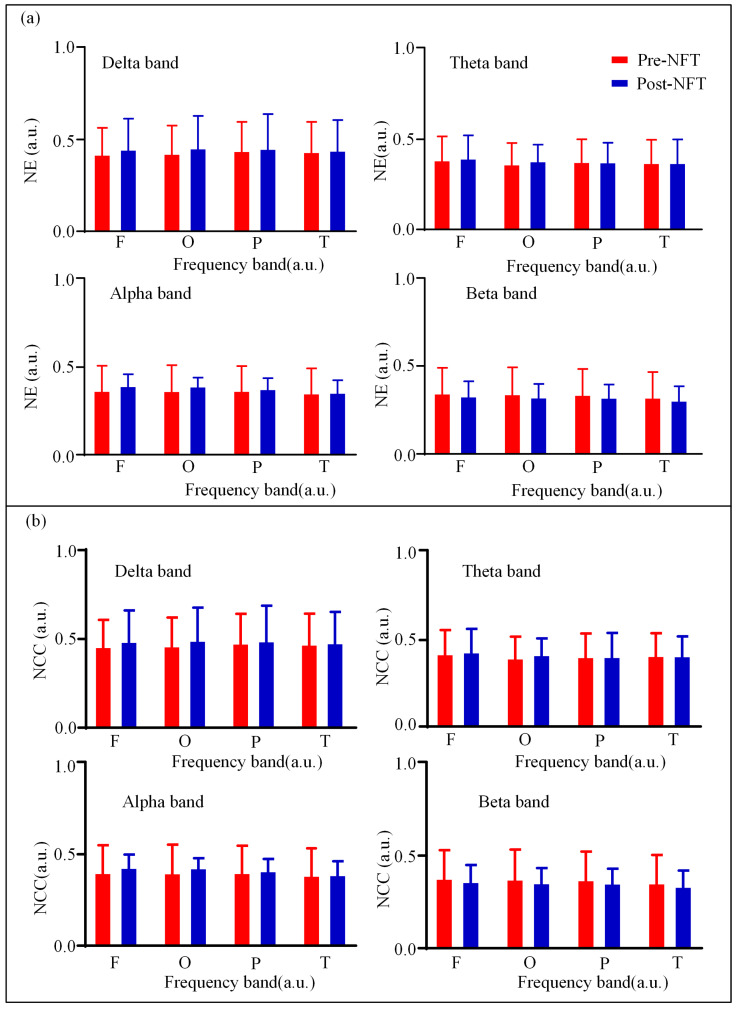
(**a**) NE and (**b**) NCC of HOFC network. Red indicates the eigenvector centrality of the LOFC network in aMCI patients before NFT, while blue indicates that after NFT.

We analyzed the differences in nodal metrics (NE; NCC) of HOFC networks across four brain regions (frontal, occipital, parietal, and temporal lobes) in different frequency bands pre- and post- NFT in individuals with aMCI. As illustrated in Figure 8, the nodal metrics of the HOFC network before NFT are depicted in red, while those after training are shown in blue. The results indicate that in the delta and alpha frequency bands, the nodal metrics across all brain regions were higher after NFT compared to those before training. In contrast, in the beta band, the nodal metrics were lower after training than before. For the theta band, the differences in nodal metrics across various brain regions were not pronounced. Furthermore, statistical comparisons of the nodal metrics between pre- and post- NFT conditions revealed no significant differences in any of the brain regions or frequency bands (*p* > 0.05). A further quantitative assessment of the nodal characteristics of the HOFC network was conducted. Detailed data for the NE and NCC across different frequency bands and brain regions are presented in Table A5, Table A6, Table A7 and Table A8, located in Appendix B.

### 3.3. Dynamic Brain Functional Network State Entropy

**Figure 9:**
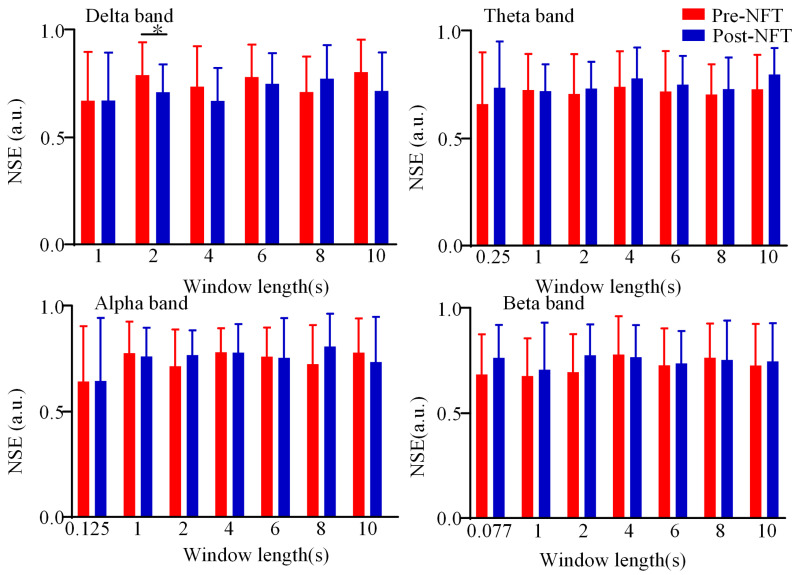
NSE of dynamic LOFC network. State entropy values pre- and post-neurofeedback training are represented by red and blue, respectively. The y-axis indicates state entropy, and the x-axis represents window length. * *p* < 0.05.

This section analyzes the differences in state entropy of the dynamic LOFC networks pre- and post- NFT in patients with aMCI across four frequency bands (delta, theta, alpha, and beta). As illustrated in Figure 9, the state entropy values pre- and post- NFT are depicted in red and blue, respectively. The vertical axis represents the state entropy, while the horizontal axis indicates the window length ($1/f_{\min}$, 1 s, 2 s, 4 s, 6 s, 8 s, and 10 s, where $f_{\min}$ denotes the minimum frequency period for each band). Statistical analysis of the differences in state entropy between pre- and post- NFT revealed no significant differences (*p* > 0.05) at small time scales (window length ≤ 1 s) across all frequency bands. A significant difference (*p* < 0.05) was observed specifically in the delta band at a window length of 2 s. No statistically significant differences were detected in the other frequency bands or at other window lengths (*p* > 0.05). Comparison of the magnitude of state entropy pre- and post- training showed that at smaller time scales, the state entropy values were generally higher after NFT. In contrast, at larger time scales, the post-training state entropy values were not consistently higher than the pre-training values, and no clear trend was observed.

**Figure 10:**
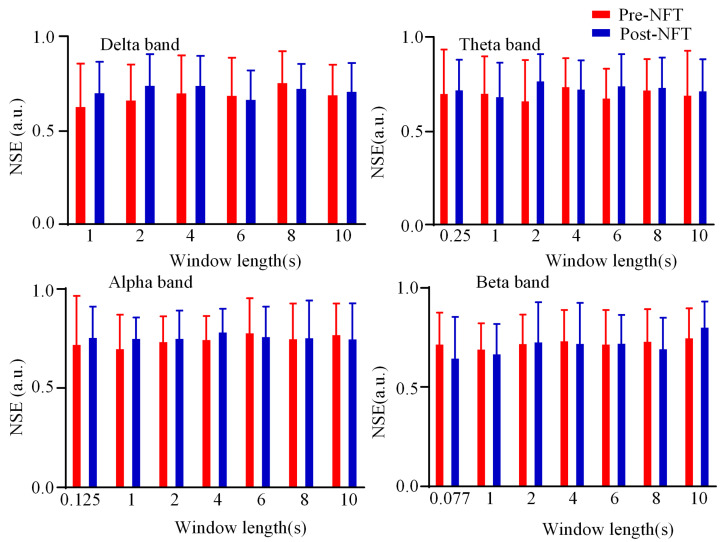
NSE of dynamic HOFC network. State entropy values pre- and post- NFT are represented by red and blue, respectively. The y-axis indicates state entropy, and the x-axis represents window length.

We analyzed the differences in state entropy of the dynamic HOFC networks pre- and post- NFT across four frequency bands (delta, theta, alpha, and beta). The corresponding box plots are presented in Figure 10. The red boxes represent the state entropy values before NFT, while the blue boxes indicate the values after training. The vertical axis corresponds to the state entropy, and the horizontal axis denotes the window length ($1/f_{\min}$, 1 s, 2 s, 4 s, 6 s, 8 s, and 10 s, where $f_{\min}$ refers to the minimum frequency period for each band). As shown in Figure 10, in the delta, theta, and alpha bands, the state entropy values after NFT were generally higher than those before training at small time scales (window length ≤ 1 s). In contrast, for the beta band, the mean state entropy after training was lower than before training at the same small time scales. Statistical comparison between pre- and post-NFT state entropy values revealed no significant differences in any of the frequency bands (*p* > 0.05).

### 3.4. Analysis of MoCA Scores

**Figure 11:**
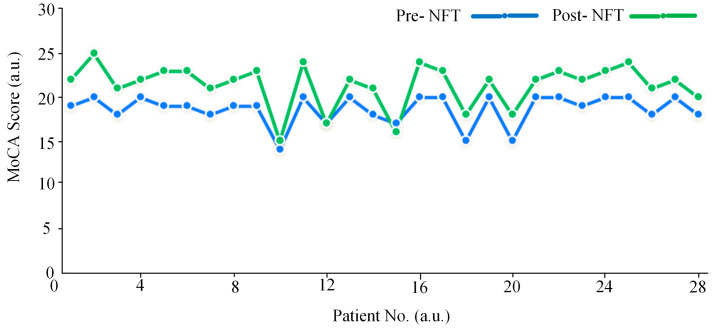
MoCA score pre- and post-NFT.

As shown in Figure 11, this study analyzed changes in MoCA scores pre- and post- NFT in 28 patients with aMCI. The results indicate that 26 patients (92.9%) exhibited a significant improvement in MoCA scores after training, one patient (3.6%) remained stable, and only one patient (3.6%) showed a decline of one point. These findings suggest that NFT has a short-term beneficial effect on cognitive function in aMCI patients, possibly mediated through mechanisms of neural plasticity. However, the long-term efficacy of this intervention requires further validation through studies with larger sample sizes and longer follow-up periods. Furthermore, the observed improvement in MoCA scores was consistent with enhanced connectivity in the delta frequency band, indicating that NFT may improve cognitive function by augmenting slow-wave synchronization.

## 4. Discussion

Through the analysis of both LOFC and HOFC networks, this study reveals the regulatory effects of NFT on brain network connectivity in patients with aMCI. The results demonstrate a significant enhancement in connectivity strength within the delta frequency band in both LOFC and HOFC networks after NFT intervention. In contrast, a general reduction in connectivity was observed in the theta, alpha, and beta bands. Dynamic network analysis indicated a significant increase in state entropy (*p* < 0.05) at a 2 s window length in the delta band for the LOFC network following NFT. Some studies have reported similar differences between healthy controls and patients with mild cognitive impairment [23,27]. Furthermore, increased state entropy under small time windows was also observed in the HOFC network across delta, theta, and alpha bands.

Clinical assessments showed that 92.9% of the subjects (26 out of 28) exhibited significant improvements in MoCA scores after NFT, with only one case showing a slight decline. This clinical outcome aligns with the observed enhancement of delta-band connectivity in the brain network analyses, supporting the hypothesis that NFT contributes to cognitive improvement through the remodeling of brain network activity [28]. Nonetheless, the lack of response or slight deterioration in a small subset of patients (3.6%) may be attributed to individual variability in responsiveness to NFT.

By restricting participants’ educational attainment to 10–15 years, this study aimed to mitigate the influence of educational level on cognitive assessments; however, this restriction introduced selection bias, thereby limiting the representativeness of the sample for the general aMCI population. In subsequent research, it will be necessary to remove the educational restriction, adopt education-adjusted versions of cognitive scales, and incorporate education as a covariate in stratified analyses in order to reduce bias [29,30].

The EEG network analysis in this study demonstrates a clear “neural mechanism–cognitive behavior” relationship with MoCA improvement: following NFT, 92.9% of patients exhibited increased MoCA scores, accompanied by enhanced static LOFC and HOFC in the delta band (1–4 Hz), rising from 0.3818 and 0.4951 to 0.4174 and 0.5112, respectively, along with elevated network efficiency in cognitive-related regions such as the frontal and parietal lobes. Delta enhancement may facilitate prefrontal–hippocampal coordination, supporting improvements in MoCA memory and executive sub-items [31], which is also corroborated by Suhail et al. [22], who confirmed its role in enhancing memory integration in MCI patients. At the dynamic level, the state entropy of delta-band LOFC networks under a 2-s window increased significantly (*p* < 0.05), reflecting im-proved flexibility in task adaptation of brain networks [32], consistent with Tazaki’s [29] assertion that dynamic network flexibility correlates with cognitive improvement. Although theta, alpha, and beta band connectivity did not show significant reduction (*p* > 0.05), suppressing their potential abnormalities may reduce neural interference [33], such as enhancing attentional control [12,34]. This pattern of “delta enhancement combined with multi-band optimization” validates the cognitive improvement mechanism underlying NFT.

The study by Lavy et al. [12] reported that only 70% of patients with MCI demonstrated improvement in memory task scores, with no significant data on overall MoCA enhancement. In contrast, the NFT implemented in our study appears to yield more clinically significant short-term cognitive improvements. Given that aMCI represents a prodromal stage of AD, the observed multidomain MoCA improvements—spanning memory, executive function, and attention suggest potential for delaying pathological progression.

Nevertheless, the durability of such short-term MoCA gains remains uncertain—a limitation pervasive in NFT research. Marlats et al. [10] observed that MoCA scores in MCI patients returned to baseline after 30 days, underscoring the potential transience of NFT effects. Although a 92.9% response rate was observed in our sample (*n* = 28), this high improvement rate may be susceptible to inflation due to limited sample size and possible outlier effects. Future studies should adopt the framework proposed by Trambaiolli et al. [30], incorporating larger cohorts, extended follow-ups (6–12 months), repeated MoCA assessments, and continuous EEG network evaluation. Employing randomized controlled trials with sham-NFT groups and independent samples *t*-tests will be essential to control for placebo effects and confirm statistical significance.

## 5. Conclusions

This study compared the strength of functional connectivity, graph-theoretical properties, and state entropy of high-order dynamic networks in patients with aMCI pre- and post- NFT. The findings are as follows: (1) NFT has induced changes in the brain network characteristics of patients with aMCI, suggesting that the brain may have undergone functional reorganization. (2) Among 28 aMCI patients, 26 showed increased MoCA scores after NFT, indicating potential beneficial effects of NFT on cognitive function in aMCI patients. (3) The long-term effects of NFT and the impacts of prolonged interventions still require further investigation.

## Figures and Tables

**Table 1:** Demographic characteristics of participants.

Characteristics	aMCI (*n* = 28)
Age (mean ± SD)	65.12 ± 4.31
Gender (male/female)	16/12
Educational level (mean ± SD)	12.63 ± 2.30
Medical history	No

Note: Medical history: neurological diseases.

## Data Availability

The data presented in this study are available on request from the corresponding author. The data are not publicly available due to ethical restrictions.

## Appendix A

A further quantitative assessment of the nodal characteristics of the LOFC network was conducted. Detailed data for the NE and NCC across different frequency bands and brain regions are presented in Table A1, Table A2, Table A3 and Table A4.

**Table A1:** Node eigenvalues of pre-and post- NFT LOFC network in delta band (mean ± SD).

	Frontal (Pre-/Post-)	Occipital (Pre-/Post-)	Parietal (Pre-/Post-)	Temporal (Pre-/Post-)
NE	0.3384 ± 0.1685/ 0.3753 ± 0.1742	0.3274 ± 0.1582/ 0.3728 ± 0.1706	0.3375 ± 0.1639/ 0.3860 ± 0.1843	0.3391 ± 0.1660/ 0.3741 ± 0.1681
NCC	0.3712 ± 0.1788/ 0.4102 ± 0.1843	0.3593 ± 0.1679/ 0.4075 ± 0.1808	0.3701 ± 0.1741/ 0.4217 ± 0.1953	0.3720 ± 0.1761/ 0.4089 ± 0.1778

**Table A2:** Node eigenvalues of pre-and post- NFT LOFC network in theta band (mean ± SD).

	Frontal (Pre-/Post-)	Occipital (Pre-/Post-)	Parietal (Pre-/Post-)	Temporal (Pre-/Post-)
NE	0.3048 ± 0.1505/ 0.3017 ± 0.1213	0.2958 ± 0.1352/ 0.2851 ± 0.0973	0.2956 ± 0.1444/ 0.2853 ± 0.1203	0.2949 ± 0.1453/ 0.2884 ± 0.1130
NCC	0.3357 ± 0.1592/ 0.3325 ± 0.1293	0.3260 ± 0.1429/ 0.3148 ± 0.1037	0.3256 ± 0.1527/ 0.3147 ± 0.1282	0.3249 ± 0.1537/ 0.3180 ± 0.1203

**Table A3:** Node eigenvalues of pre-and post- NFT LOFC network in alpha band (mean ± SD).

	Frontal (Pre-/Post-)	Occipital (Pre-/Post-)	Parietal (Pre-/Post-)	Temporal (Pre-/Post-)
NE	0.3079 ± 0.1461/ 0.3074 ± 0.0888	0.2996 ± 0.1347/ 0.2999 ± 0.0881	0.2920 ± 0.1398/ 0.2768 ± 0.0727	0.2902 ± 0.1339/ 0.2786 ± 0.0737
NCC	0.3394 ± 0.1546/ 0.3394 ± 0.0952	0.3305 ± 0.1425/ 0.3313 ± 0.0945	0.3220 ± 0.1479/ 0.3061 ± 0.0779	0.3202 ± 0.1414/ 0.3080 ± 0.0789

**Table A4:** Node eigenvalues of pre-and post- NFT LOFC network in beta band (mean ± SD).

	Frontal (Pre-/Post-)	Occipital (Pre-/Post-)	Parietal (Pre-/Post-)	Temporal (Pre-/Post-)
NE	0.2236 ± 0.1580/ 0.2089 ± 0.0625	0.2188 ± 0.1535/ 0.2001 ± 0.0551	0.2305 ± 0.1555/ 0.2061 ± 0.0750	0.2167 ± 0.1505/ 0.1965 ± 0.0637
NCC	0.2491 ± 0.1672/ 0.2339 ± 0.0671	0.2440 ± 0.1624/ 0.2244 ± 0.0591	0.2567 ± 0.1646/ 0.2309 ± 0.0807	0.2415 ± 0.1593/ 0.2202 ± 0.0685

## Appendix B

A further quantitative assessment of the nodal characteristics of the HOFC network was conducted. Detailed data for the NE and NCC across different frequency bands and brain regions are presented in Table A5, Table A6, Table A7 and Table A8.

**Table A5:** Node eigenvalues of pre-and post- NFT HOFC network in delta band (mean ± SD).

	Frontal (Pre-/Post-)	Occipital (Pre-/Post-)	Parietal (Pre-/Post-)	Temporal (Pre-/Post-)
NE	0.4200 ± 0.1435/ 0.4474 ± 0.1668	0.4247 ± 0.1518/ 0.4547 ± 0.1745	0.4394 ± 0.1565/ 0.4509 ± 0.1877	0.4338 ± 0.1623/ 0.4409 ± 0.1656
NCC	0.4559 ± 0.1520/ 0.4851 ± 0.1765	0.4603 ± 0.1609/ 0.4922 ± 0.1851	0.4766 ± 0.1658/ 0.4889 ± 0.1991	0.4706 ± 0.1721/ 0.4779 ± 0.1752

**Table A6:** Node eigenvalues of pre-and post- NFT HOFC network in theta band (mean ± SD).

	Frontal (Pre-/Post-)	Occipital (Pre-/Post-)	Parietal (Pre-/Post-)	Temporal (Pre-/Post-)
NE	0.3851 ± 0.1312/ 0.3958 ± 0.1265	0.3631 ± 0.1161/ 0.3808 ± 0.0896	0.3703 ± 0.1278/ 0.3705 ± 0.1293	0.3760 ± 0.1242/ 0.3749 ± 0.1062
NCC	0.4192 ± 0.1388/ 0.4306 ± 0.1344	0.3957 ± 0.1236/ 0.4147 ± 0.0951	0.4031 ± 0.1349/ 0.4035 ± 0.1376	0.4092 ± 0.1304/ 0.4082 ± 0.1128

**Table A7:** Node eigenvalues of pre-and post- NFT HOFC network in alpha band (mean ± SD).

	Frontal (Pre-/Post-)	Occipital (Pre-/Post-)	Parietal (Pre-/Post-)	Temporal (Pre-/Post-)
NE	0.3662 ± 0.1419/ 0.3934 ± 0.0665	0.3651 ± 0.1462/ 0.3912 ± 0.0494	0.3523 ± 0.1405/ 0.3558 ± 0.0695	0.3666 ± 0.1399/ 0.3759 ± 0.0609
NCC	0.3992 ± 0.1501/ 0.4281 ± 0.0708	0.3978 ± 0.1548/ 0.4258 ± 0.0530	0.3842 ± 0.1486/ 0.3876 ± 0.0743	0.3995 ± 0.1478/ 0.4092 ± 0.0651

**Table A8:** Node eigenvalues of pre-and post- NFT HOFC network in beta band (mean ± SD).

	Frontal (Pre-/Post-)	Occipital (Pre-/Post-)	Parietal (Pre-/Post-)	Temporal (Pre-/Post-)
NE	0.3455 ± 0.1442/ 0.3289 ± 0.0843	0.3417 ± 0.1507/ 0.3231 ± 0.0746	0.3222 ± 0.1433/ 0.3049 ± 0.0800	0.3380 ± 0.1448/ 0.3213 ± 0.0729
NCC	0.3775 ± 0.1524/ 0.3594 ± 0.0901	0.3733 ± 0.1595/ 0.3532 ± 0.0798	0.3522 ± 0.1514/ 0.3337 ± 0.0857	0.3693 ± 0.1530/ 0.3514 ± 0.0781
